# Affordable diagnosis and prevention of genetic disease

**DOI:** 10.1186/1755-8166-7-S1-I6

**Published:** 2014-01-21

**Authors:** John Burn

**Affiliations:** 1 Institute of Genetic Medicine, Newcastle University, International Centre for Life, Newcastle upon Tyne, UK

## 

Pathogenic variants in around 100 genes are responsible for a high risk of early onset solid tumours in close to 1% of the world’s population. As sequencing costs plummet and effective drugs are targeted at the molecular structure of cancers, many more of these gene carriers will be identified. They are a target population for low cost prevention strategies in their own right. An added benefit is that similar molecular pathways are disrupted in sporadic tumours, so effective chemoprevention strategies can be extrapolated to the general population. The Cancer Prevention Programme, CaPP, was developed 25 years ago to promote genetically targeted cancer prevention trials. CAPP2, published in 2011 (Burn et al Lancet) was the first trial with cancer as an endpoint to prove that regular aspirin significantly reduced the burden of colorectal and other cancers in people at risk of Lynch syndrome due to a defective mismatch repair gene. 600mg daily (2 tablets) for 2 years or more than halved the cancer rate after a lag of five years. Long term follow up of the other trial which tested cancer as a cancer preventive, the Women’s Health Study, has now shown a protective effect of very low dose aspirin taken on alternate days, but not until 10 years from the trial start (Cook et al 2013). CaPP3 starts in 2014 and will test the relative benefits of three different blinded doses of enteric coated aspirin -600mg, 300mg and 100mg in 3000 gene carriers. Failure of mismatch repair leads to the generation of frameshift peptides due to unrepaired slippage in repetitive DNA stretches (microsatellites). Antibodies to the resulting frame shift peptides will be tested as possible biomarkers. A vaccine raised against the important frame shift peptides affecting genes involved in carcinogenesis is also under investigation.

Our novel nanowire technology is being investigated as a means of identifying microsatellite instability and BRAF mutations which help separate sporadic from hereditary tumours. This will make case identification much cheaper and easier in the context of countries with a developing economy.

